# Coupling Effects of Stress and Carbonation on Concrete Durability: A Review

**DOI:** 10.3390/ma17225438

**Published:** 2024-11-07

**Authors:** Zhixin Liu, Chaochao Sun, Jili Qu

**Affiliations:** 1 School of Environment and Architecture, University of Shanghai for Science and Technology, Shanghai 200093, China; 232291893@st.usst.edu.cn; 2 School of Civil Engineering, Kashi University, Kashi 844000, China; 17799770889@163.com

**Keywords:** concrete, stress, carbonization, fly ash

## Abstract

This review investigates the combined effects of stress and carbonation on the durability of concrete, an important but under-researched factor affecting infrastructure longevity. Carbonation substantially degrades concrete, particularly under tensile, compressive, and bending stresses. This paper synthesizes recent findings to explore how these stress states influence the progression of carbonation and overall durability, emphasizing data on carbonation depth, mechanical performance, and structural integrity. Key models and experimental results are evaluated, revealing significant gaps in current knowledge, including limited insights into the long-term impacts of stress-carbonation interactions and the lack of standardized testing methods. To address these gaps, future research should prioritize the refinement of carbonation prediction models under complex stress conditions and the development of high-resilience materials suitable for challenging environments. Ultimately, this review aims to establish a foundation for more accurate predictions of concrete service life, thereby supporting advancements in material science and sustainable construction practices.

## 1. Introduction

Carbon dioxide (CO_2_) is a major component of global greenhouse gases, and its emissions have profoundly affected climate change and environmental pollution. According to the IEA, global CO_2_ emissions have risen substantially over the past few decades, primarily due to energy consumption, transportation, and industrial activities [1]. The energy sector is responsible for nearly two-thirds of total emissions, with power and heat production being the most significant contributors, accounting for approximately 42% of global emissions [2]. Emissions from the transportation sector primarily result from fuel consumption, constituting approximately 24% of global CO_2_ emissions [3,4]. Industrial emissions largely originate from the cement, steel, and chemical industries, with cement production alone contributing 8% of global emissions [5,6]. Energy consumption and associated emissions from buildings are considerable, accounting for approximately 30% of the total energy use [7].

**Figure 1:**
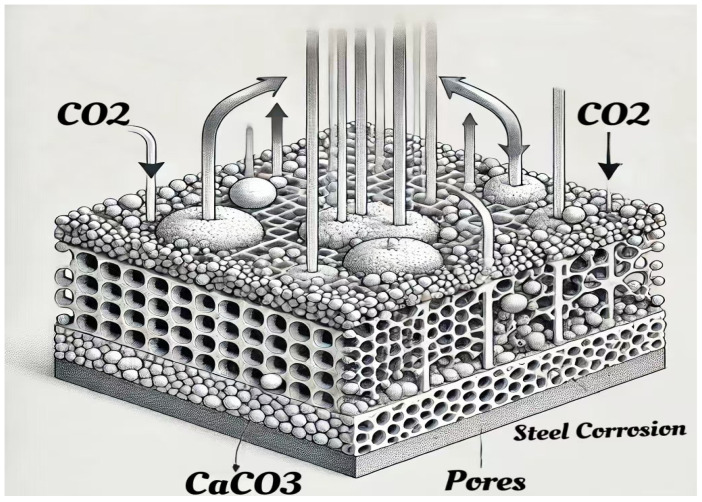
Carbonization of concrete and corrosion of steel bars.

The increasing concentration of CO_2_ in the atmosphere has positioned concrete carbonation as a critical area of research regarding the durability of engineering structures As illustrated in Figure 1, concrete carbonation occurs when atmospheric CO_2_ penetrates the porous structure of concrete, reacting with alkaline compounds, primarily calcium hydroxide and a minor amount of hydrated calcium silicate, to form calcium carbonate (CaCO_3_) [8,9,10,11,12,13,14]. This reaction reduces the alkalinity of concrete; once the pH drops to 9–11, the protective passivation layer on reinforcing steel bars is compromised, rendering them susceptible to corrosion [15,16]. Mehta [17,18] emphasized in his review of concrete durability research that steel corrosion resulting from carbonation is a significant contributor to concrete deterioration. Newly poured concrete primarily relies on alkaline hydration products, maintaining a pH of approximately 12.5 near the reinforcing steel. This elevated pH facilitates the formation of a dense passivation layer on the steel surface, providing effective protection. However, in a typical atmospheric environment, the ingress of H_2_O and CO_2_, along with reactions with alkaline hydration products, gradually reduces the pH, a process referred to as concrete carbonation [19]. As concrete ages and undergoes cracking under various loading conditions, a range of substances can infiltrate through the pores and fissures of the concrete over time. These substances chemically interact with the materials within the concrete, resulting in a decrease in the pH surrounding the reinforcement. This decline in pH contributes to the degradation of the passive film on the steel surface, thereby accelerating reinforcement corrosion [20].

As corrosion begins, the volume of the steel increases, creating significant tensile stresses within the concrete protective layer, potentially leading to spalling. This spalling exacerbates crack propagation and diminishes the effective bearing area of the concrete, ultimately reducing its load-bearing capacity and initiating a detrimental cycle [18]. This process adversely affects the durability of concrete structures and is closely associated with the safety and longevity of large-scale engineering projects [20]. For instance, high-rise buildings in São Paulo, Brazil, have exhibited cracking and spalling due to carbonation-induced reinforcement corrosion, posing a risk of collapse [21]. In Barcelona, Spain, road and bridge structures have experienced severe corrosion due to concrete carbonation. A parking structure in Sydney, Australia, experienced multiple cracks attributed to carbonation [22], significantly impacting its safety [23]. Furthermore, in Milan, Italy, long-term neglect and worsening carbonation resulted in severe reinforcement corrosion in a residential building, ultimately causing the collapse of several floor slabs [24,25].

In addition, the issue of carbonation has led to substantial economic losses in the concrete sector both domestically and internationally. Europe has allocated approximately 50% of its infrastructure budget to prevent and repair concrete corrosion [9]. In 2002, the United States incurred economic costs amounting to approximately 155 billion US dollars for the repair of corroded structures [10]. In 2014, China’s expenditures related to this issue reached as high as 3.34% of its GDP [11]. Moreover, these economic losses are exacerbated by the proliferation of aging concrete buildings and increasing atmospheric CO_2_ levels. For instance, in Hong Kong, China, the number of residential buildings exceeding 50 years old surpassed 30,000 in 2017 and rapidly increased to over 60,000 by 2020. It can be predicted that the carbonization problem of old buildings in developed countries such as Europe and the United States will be more serious.

The presence of cracks creates pathways for corrosive agents, such as external CO_2_, to infiltrate the concrete interior, thereby accelerating the carbonation process. Cracks are particularly likely to develop when concrete is subjected to complex stress conditions, which further deteriorate its structural performance [26]. Therefore, it is essential to study the carbonation resistance of concrete under various loading conditions, as well as the combined effects of stress and carbonation on structural durability. Effectively designing durable structures, particularly for specific service life and operational conditions, necessitates accurate predictions of the depth and rate of concrete carbonation, which has become a central focus of current research [27].

Despite substantial advancements in understanding concrete carbonation over the past few decades, relatively few studies have systematically investigated the effects of various stress states on carbonation rates and depths. Furthermore, limited research has examined the influence of fly ash and stress conditions on carbonation. Most codes classify the concrete carbonation environment and the resultant steel bar corrosion as the general environmental conditions, categorizing their corresponding effect level as slight, mild, and moderate, while other environmental effects are deemed serious or very serious [28,29,30]. Given the widespread use of prestressed concrete and the diverse loads that concrete structures endure during service, understanding the impact of stress on the carbonation process is crucial for extending the lifespan and maintaining engineering structures. It is also essential to optimize fly ash concrete to mitigate carbonation and to develop more accurate long-term carbonation models under varying stress conditions.

This article presents a comprehensive literature review using databases such as Web of Science, CNKI, Google Scholar, and Scopus, focusing on research published from 1994 to 2024. The review prioritizes studies that offer experimental and theoretical insights, particularly those investigating the coupling effect of stress and carbonation. These studies are assessed based on their methodologies, results, and relevance to contemporary engineering practices to establish a general framework. Distinct keyword searches ensure that substantial data underpins each influencing factor.

**Figure 2:**
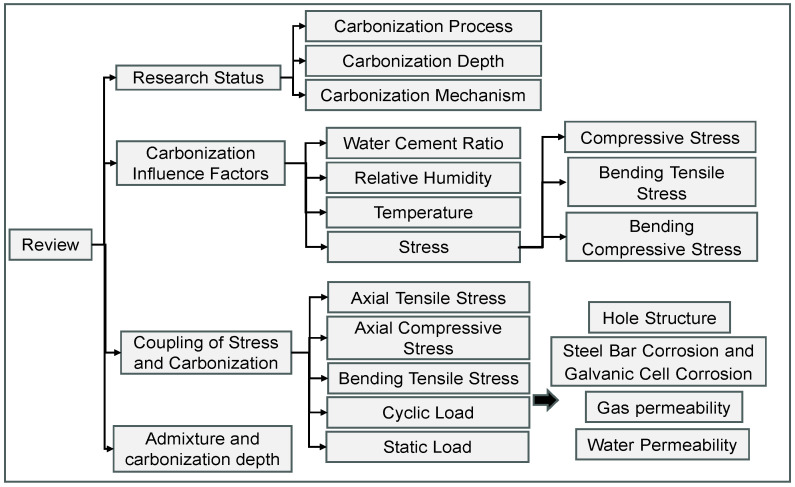
Overview diagram.

A systematic review of the carbonation mechanism is presented, emphasizing the summarization of test studies under various stress states, including tensile, compressive, bending, and cyclic loads. Additionally, the review investigates the effects of concrete cracks, permeability, different aggregates, and steel corrosion on carbonation performance. It also analyzes the durability of concrete under the combined effects of multiple stresses and carbonation. Finally, strategies for mitigating the effects of stress and carbonation on concrete are examined in detail. Figure 2 illustrates the framework of this review.

## 2. Research Status and Main Point of View

### 2.1. Research Status of the Concrete Carbonization Process

**Figure 3:**
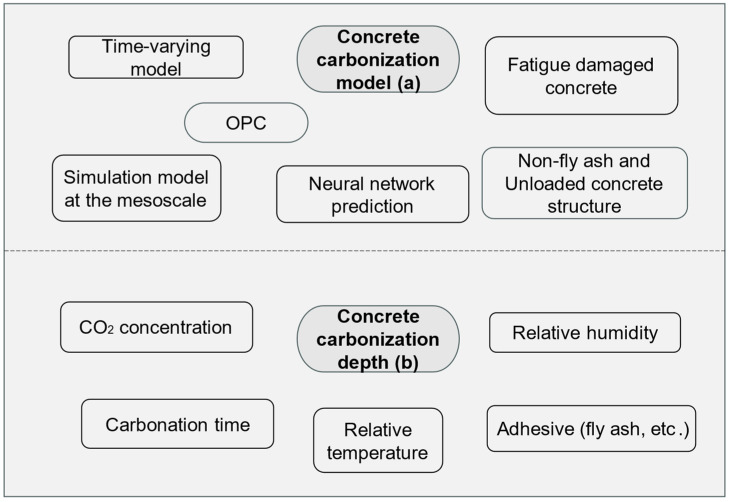
Research status map: (**a**) Concrete carbonization model [31,32,33,35,36,40]; (**b**) Concrete carbonization depth [31,34,35,36,37,39,40].

Carbonation significantly reduces the alkalinity of concrete, resulting in the corrosion of steel reinforcement, which is a critical factor to consider in the durability design of concrete structures. It is essential to consider carbonation depth in such designs, as it directly impacts the longevity and integrity of the structure. While numerous experimental studies have investigated rapid carbonation, their contributions differ in scope and applicability. Figure 3 summarizes the current state of research in recent years.

Ishida et al. (2008) [31] developed a thermo-hygro-physical model that accurately predicts carbonation depth and microstructural changes under various environmental conditions. However, the limitation of the model lies in its failure to account for micropore variations, which restrict its applicability to ordinary Portland cement. This highlights a gap in current models, indicating the need for a more comprehensive approach that incorporates micropore dynamics for broader applicability. X. Ruan et al. (2012) [32] introduced a mesoscale numerical model based on mass conservation and chemical kinetics, revealing that both maximum aggregate size and volume fraction significantly affect carbonation rates. This finding underscores the significance of material composition in mitigating carbonation effects. Talukdar et al. (2012) [33] further advanced the field by creating a carbonation model that considers time-varying CO_2_ concentrations, temperature, and humidity, thereby enhancing predictive capabilities for non-pozzolanic, non-load-bearing concrete structures. This adaptability is crucial because it mirrors real-world conditions, indicating that future models should similarly incorporate varying environmental factors.

Cui et al. (2015) [34] examined the effects of CO_2_ concentration on carbonation depth, noting that carbonation depth increases rapidly below 20% CO_2_ concentration, while the rate diminishes above this threshold. This indicates that optimal CO_2_ levels are critical for accelerating carbonation, which could inform the design of more resilient concrete structures. Li et al. (2017) [35] explored the impact of organic film coatings on carbonation resistance, revealing that these coatings significantly extend the service life of concrete. This suggests a promising avenue for enhancing concrete durability through protective measures.

Jiang et al. (2017) [36] investigated the impact of organic film coatings on carbonation resistance, revealing that these coatings significantly extend the service life of concrete. This points to a promising avenue for enhancing concrete durability through protective measures. Chen et al. (2018) [37] demonstrated that carbonation depth correlates linearly with temperature and adheres to distinct functions concerning CO_2_ concentration and humidity. These insights reveal the complex interplay between environmental factors and carbonation, necessitating future studies to further explore these relationships.

Zhou et al. (2019) [38] observed that carbonation depth in coal mine environments varied significantly over time, indicating that exposure conditions play a crucial role in carbonation kinetics. Zhang et al. (2020) [39] examined the effects of carbonation time and fly ash content, establishing a single-factor carbonation model that emphasizes the role of supplementary cementitious materials in enhancing concrete performance. Most recently, Huo et al. (2023) [40] employed an inverse variance method combined with artificial neural networks to predict carbonation depth, identifying carbonation time, CO_2_ concentration, and binder dosage as critical factors. This innovative approach illustrates the potential of machine learning to improve predictive models.

In summary, while significant advancements have been made in understanding carbonation, there remains a pressing need for integrated models that account for micropore variations, environmental dynamics, and material properties. Future research should concentrate on developing standardized testing methods and innovative modeling techniques that reflect the complexities of real-world conditions, ultimately leading to more durable concrete structures.

### 2.2. The Current Status of Research on the Impact of Concrete Stress on Carbonation Depth

Concrete structures are typically subjected to various loads, which distinctly influence their durability compared to unstressed concrete. Consequently, a growing body of research has focused on understanding how loading or stress affects carbonation depth in concrete.

Tian et al. (2010) [41] conducted accelerated carbonation tests, finding that tensile stress accelerated the carbonation rate while compressive stress slowed it down. They noted that the magnitude of stress change correlates directly with the carbonation rate, emphasizing the need to account for stress variations in durability assessments. Zheng (2013) [42] explored the anti-carbonation performance of self-compacting concrete across various stress levels and proposed a predictive method for carbonation depth under stress conditions, reinforcing the need for tailored design approaches in structural applications. Ruan et al. (2013) [43] quantitatively evaluated the impact of stress on carbonation within concrete cross-sections and corners, developing a two-dimensional numerical model that highlighted accelerated carbonation at corners due to enhanced CO_2_ diffusion. This finding suggests that structural design should consider both geometry and stress distribution to mitigate the effects of carbonation. Jiang et al. (2015) [44] examined the carbonation process of concrete beams under cyclic loads, introducing a model that encompasses nine key factors, including cement content and residual strain, which influence carbonation depth in fatigue-damaged beams. Their research underscores the complex interplay between mechanical loading and chemical processes, warranting further investigation.

Sun et al. (2015) [45] discussed the coupling effects of axial compression load and carbonation on the permeability of chloride ions, gases, and water, finding that air permeability decreased as carbonation progressed under load. This relationship is crucial, indicating that carbonation can potentially enhance the durability of concrete by reducing permeability to harmful agents. Liang et al. (2016) [46] conducted cyclic and monotonic loading tests on concrete prisms, discovering that as carbonation depth increased, peak strain slightly decreased, peak stress increased, and ultimate strain decreased. This indicates a need for a nuanced understanding of how carbonation affects material performance under varying loading scenarios. Liu et al. (2019) [47] investigated the effects of fly ash content and bending load on carbonation performance in reinforced concrete, finding that higher fly ash content increased carbonation susceptibility to carbonation. This suggests that while supplementary cementitious materials can enhance concrete properties, their interactions with carbonation must be managed carefully over time.

Liu (2019) [48] proposed a “carbonation influence coefficient” to study the relationship between carbonation in the bending-tension and bending-compression zones, providing a valuable tool for assessing carbonation damage in practical projects. This innovative approach establishes a scientific basis for preventive measures in structural design. Miao (2019) [49] conducted flexural fatigue tests on concrete subjected to vehicle loads, demonstrating that carbonation depth adhered to Fick’s first law and increased with carbonation time. This resulted in the development of a durability prediction model for vehicle-loaded concrete under combined carbonation and fatigue loads, highlighting the need for integrated models that reflect real-world conditions.

Cao (2021) [50] examined the effects of freeze-thaw cycles on fully carbonized concrete, noting that both axial tensile and compressive stresses increased after carbonation, suggesting that the environmental conditions further complicate the carbonation process. Zheng (2022) [51] applied deep learning to investigate the mechanical and carbonation properties of mixed fiber iron tailings concrete, establishing a carbonation depth equation. The addition of fibers improved the concrete’s strength, elastic modulus, peak strain, and carbonation resistance. Chen (2022) [52] investigated the effects of concrete strength, curing age, and loads on chloride ion diffusion, concluding that carbonation accelerates chloride transport, raising concerns for long-term durability. Zheng (2022) [53] further explored the impact of iron tailing replacement rates and fiber content on mechanical properties, carbonation depth, and porosity, finding that a 30% replacement rate optimized strength while minimizing porosity.

Long (2023) [54] introduced a transient pore water saturation equation into the classical carbonation kinetic model, demonstrating that increased pore water saturation reduced CO_2_ diffusivity and enhanced carbonation resistance. Wang (2023) [55] employed the Split Hopkinson Pressure Bar (SHPB) to conduct dynamic uniaxial compression tests on samples with varying carbonation ages. The results indicated that peak stress increased with impact pressure, suggesting an apparent strain rate effect.

In summary, over the past decade, researchers have conducted numerous rapid carbonation tests and proposed various carbonation models, refining factors such as carbonation and load influence coefficients to enhance model accuracy. Several studies have also focused on addressing carbonation issues in fly ash concrete by reducing carbonation depth through additives such as iron tailings and basalt fibers, thereby enhancing the durability of concrete under load.

### 2.3. Carbonization Mechanism of Concrete

**Figure 4:**
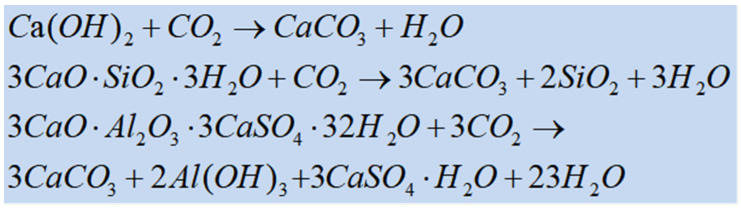
Carbonation equation [56].

The alkaline environment within the concrete structure contributes to the formation of the passivation film on the steel bar. This process eventually leads to the loss of passivation of steel bars and accelerated corrosion, while the concrete experiences shrinkage and increased brittleness. The specific carbonation equation is illustrated in the Figure 4 [56].

## 3. Factors Affecting Carbonation of Concrete

Factors influencing carbonization can be categorized into internal and external categories. Internal factors pertain to the material aspects of concrete mix design, encompassing parameters such as the water-cement ratio, cement dosage, curing conditions, and concrete strength. Conversely, external factors are associated with the service conditions of concrete, such as relative humidity, temperature, CO_2_ concentration, and concrete stress state.

### 3.1. Effect of Water-Cement Ratio on Concrete Carbonation

As shown in Table 1, whether it is Ordinary Portland Cement Concrete (OPC) or Slag Cement Concrete (PSC), the carbonization depth of concrete increases with the water-cement ratio [57,58,59,60,61].

### 3.2. Effect of Relative Humidity on Concrete Carbonization

When relative humidity is low, carbonation is hindered by the absence of a liquid-phase environment. Conversely, when humidity is high, excessive moisture content in the concrete can impede the diffusion of carbon dioxide, thus slowing the carbonation rate. Research indicates that the carbonation rate of concrete decreases with increasing relative humidity. Ji [62] found that the carbonation coefficient exhibits a parabolic relationship with relative humidity (RH). Specifically, when RH is between 40% and 70%, the carbonation rate of concrete tends to increase. Du [63] suggests that the carbonation rate reaches its peaks when RH is between 50% and 60%. The currently accepted formula for the influence of relative humidity on carbonation is presented in Equation (1) [64].

(1) $$\frac{k_{RH_{1}}}{k_{RH_{2}}}=\frac{{\left(1-RH_{1}\right)}^{1.1}}{{\left(1-RH_{2}\right)}^{1.1}}$$

RH is the relative humidity of the ITH environment.

**Figure 5:**
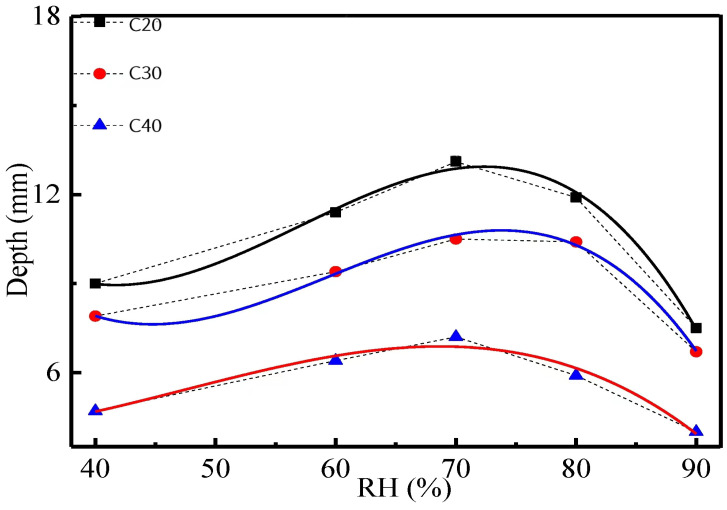
Impact of relative humidity on carbonization depth [37].

Chen [37] studied and simulated the carbonization depth of C20, C30, and C40 concrete under different humidity, and the results obtained are shown in Figure 5.

According to the research results of Ji [62], Du [63], Jiang [64], Chen [37], and Li [65], it is evident that as relative humidity increases, the carbonation depth first becomes more extensive and then diminishes.

### 3.3. Effect of Temperature on Carbonization

The influence of temperature is primarily related to the carbonation rate. The higher the temperature, the faster the rate of carbonation. However, as the temperature rises, the solubility of carbon dioxide and calcium hydroxide decreases, inhibiting the carbonization reaction [66]. Loo [67] concluded that the temperature’s effect was negligible between 20 °C and 40 °C. However, it has also been proposed that the carbonation reaction is intensified when temperature increases from 20 °C to 40 °C. According to the carbonation database, Tsinghua University has provided the formula (2) that describes the influence of temperature on carbonation:

(2) $$\frac{k_{T_{1}}}{k_{T_{2}}}={\left(\frac{T_{1}}{T_{2}}\right)}^{\frac{1}{4}}$$

T_j_ is the temperature of the JTH environment, and K_T_ is obtained from the Uomoto Kenichi model [68] and Niu Di-tao model [69].

**Figure 6:**
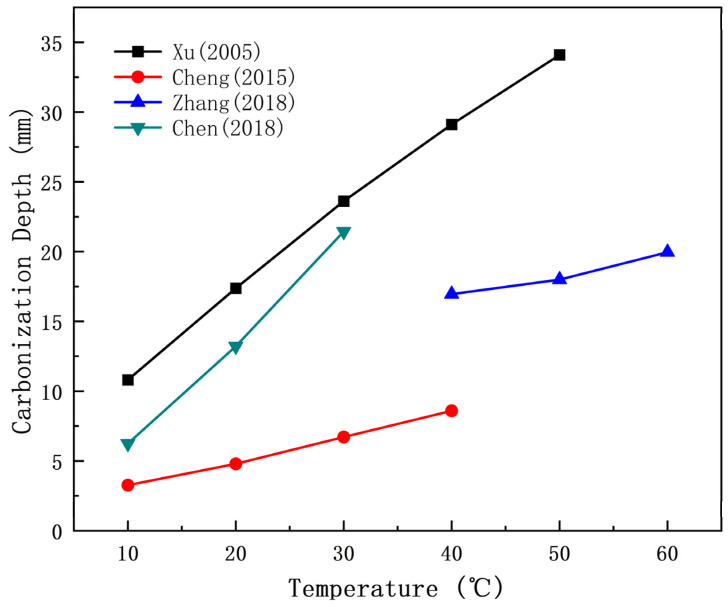
Relationship between temperature and carbonation depth [37,70,71,72].

The results of Chen [37], Xu [70], Cheng [71], and Zhang [72] are shown in Figure 6.

Figure 6 illustrates that the carbonation depth of concrete increases with the temperature within a specific range.

### 3.4. Effect of Stress on Concrete Carbonization

Castel [73] placed two concrete beams in an atmosphere environment for 13 years while simultaneously applying a bending load to both beams to investigate the relationship between carbonation depth and tensile stress. The results indicate that the applied load and the density of the concrete significantly influence CO_2_ infiltration into the material.

(3) $$CD\left(\sigma_{0}\right)=CD_{0}g\left[1+\alpha_{g}{\left(\frac{h_{g}\sigma_{s}}{d}\right)}^{3}\right]$$

Tu Yongming [74] investigated the durability of concrete structures under external tensile and compressive stresses. The results demonstrated that tensile stress accelerated the carbonation rate of concrete; the greater the tensile stress, the higher the carbonation rate. Conversely, increased compressive stress was correlated with a reduced carbonation rate.

(4) $$x=\eta k_{R_{h}}k_{T}k_{co_{2}}k_{we}x_{\sigma }\sqrt{t}$$

The empirical carbonation depth model is outlined in CECS 220:2007 [75]. This model primarily considers the effects of concrete compressive strength, ambient temperature, relative humidity, CO_2_ concentration, and stress within the concrete on carbonation depth.

(5) $$x=K\sqrt{t}$$

Building on the existing carbonation empirical model, Wang et al. [76] introduced the stress influence coefficient and quantitatively examined the relationship between this coefficient and the stress ratio in concrete. They provided a corresponding relationship formula. The results indicate that the stress influence coefficient increases with the tensile stress ratio of concrete, while it decreases as the compressive stress ratio increases.

(6) $$x_{\sigma }=(1.00+0{.40\delta }_{T}+0{.270\delta }_{T}^{2}{)x}_{0}$$

(7) $$x_{\sigma }=\left(1.00-0.30\delta_{c}+0.16\delta_{c}^{2}\right)x_{0}$$

To investigate the neutral depth of concrete members affected by carbonation, acid rain, stress, and other individual factors, as well as the coupling of multiple factors, Xu et al. [77] considered the influence of tensile and compressive stress within a specified range.

(8) $$k_{\sigma }=1+0.15S_{t}^{0.71}$$

(9) $$k_{\sigma }=1-0.87S_{c}^{0.69}$$

Sun [78] introduced the growth rate of carbonation depth to better analyze the influence of the freeze-thaw cycle on concrete carbonization.

(10) $$\eta_{i}=(x_{i}-x_{k})/x_{i}$$

1. CD: concrete carbonization depth; σs: tensile stress of concrete; CD_0_: carbonation depth at 20 cm of the concrete sample without bending load; hσs/d: effective tensile stress;
2. η = 1.6175; k_Rh_: Influence coefficient of environmental relative humidity; k_T_: environmental temperature influence coefficient;
3. x represents the depth of carbonization
4. x_σ_: carbonization depth of concrete under stress: carbonation depth under zero stress: tensile stress ratio;
5. The application range of St is 0~0.8; The application range of Sc is 0~0.3.
6. x_i_ is the carbonization depth under the combined action of freeze-thaw carbonization, x_k_ is the concrete carbonization depth of pure carbonization.

#### 3.4.1. Effect of Compressive Stress on Concrete Carbonization

When the compressive stress zone of a beam is subjected to loading, microcracks within and on the surface tend to close or diminish, hindering the infiltration of external CO_2_ and water. Over time, the cement hydration products in the concrete surface layer undergo carbonation due to the infiltration of CO_2_ and water. The byproducts generated during this process fill the pores, further restricting the infiltration of CO_2_ and water.

**Figure 7:**
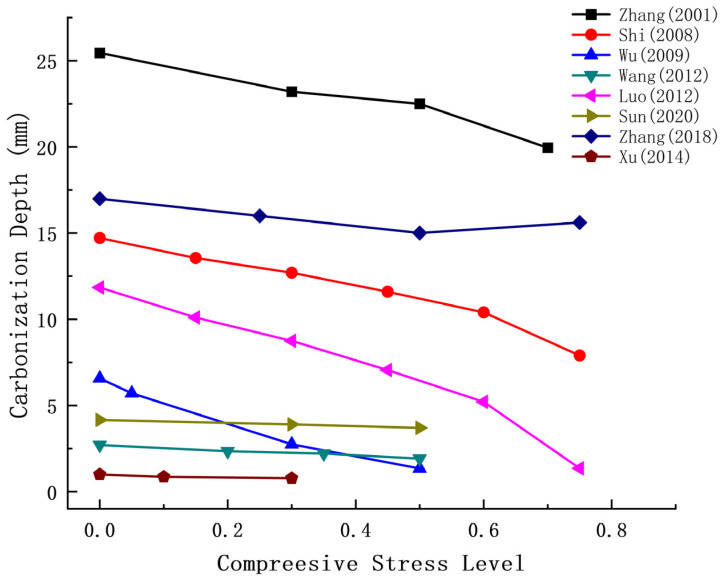
Relationship between compressive stress and carbonation depth [72,77,78,81,82,83,84,85].

Current research on compressive stress indicates that at a compressive stress level of 0.3 fc, the carbonation depth of ordinary concrete and fly ash concrete specimens is smaller than that of the corresponding unstressed specimens. However, when the compressive stress exceeds 0.3 fc, the carbonation depth increases with the increasing stress level. Several researchers have reached similar conclusions. Wan et al. [79] demonstrated that the carbonation depth of concrete is minimized at a stress level of 0.35 to 0.65 times its compressive strength. Tang et al. [80] proposed that when the peak compressive stress exceeds 0.2 times the compressive strength, the carbonation depth decreases with increasing compressive stress, revealing a different trend. Therefore, 0.2 times the peak compressive strength of concrete is considered a critical value, applicable even under cyclic loading. Additional research data are presented in Table 2 and Figure 7.

As shown in Figure 7, most researchers’ data indicate that the carbonization depth of concrete decreases with increasing compressive strength. However, Zhang’s research [72] identifies a cut-off point at 0.5 fc, beyond which the carbonation depth increases again.

#### 3.4.2. Effect of Flexural Tensile Stress on Concrete Carbonization

**Figure 8:**
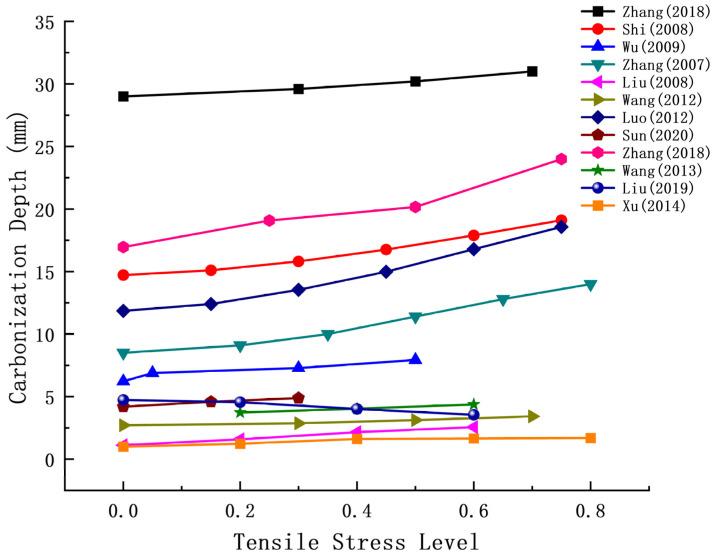
Relationship between flexural and tensile stress and carbonation depth [48,72,77,78,81,82,83,84,85,86,87,88].

Lu Xiangyu et al. [86] demonstrated that the stress gradient in the tensile stress zone of a concrete beam subjected to bending loads is relatively large, with the maximum tensile strain being 2 to 4 times greater than that observed in axial tensile specimens. Zhang Wei, Chen Feng, Guo Qingxing, and colleagues investigated the one-dimensional and two-dimensional carbonation behaviors of fly ash concrete under various bending tensile stress levels. Their findings indicated that concrete carbonation was significantly accelerated when subjected to bending tensile stress. Additional research data can be found in Table 3 and Figure 8.

Most research findings indicate that the carbonation depth of concrete increases with rising tensile stress levels due to bending load. The presence of bending and tensile stresses facilitates the infiltration of CO_2_ into the interior of the concrete, exacerbating carbonation damage, particularly in the tensile zone. Under the same fly ash content, flexural loads significantly influence the carbonation of reinforced concrete blocks. Some researchers have identified an exponential relationship between the influence coefficient of bending load on carbonation and the bending stress level.

#### 3.4.3. Effect of Bending Compressive Stress on Concrete Carbonization

**Figure 9:**
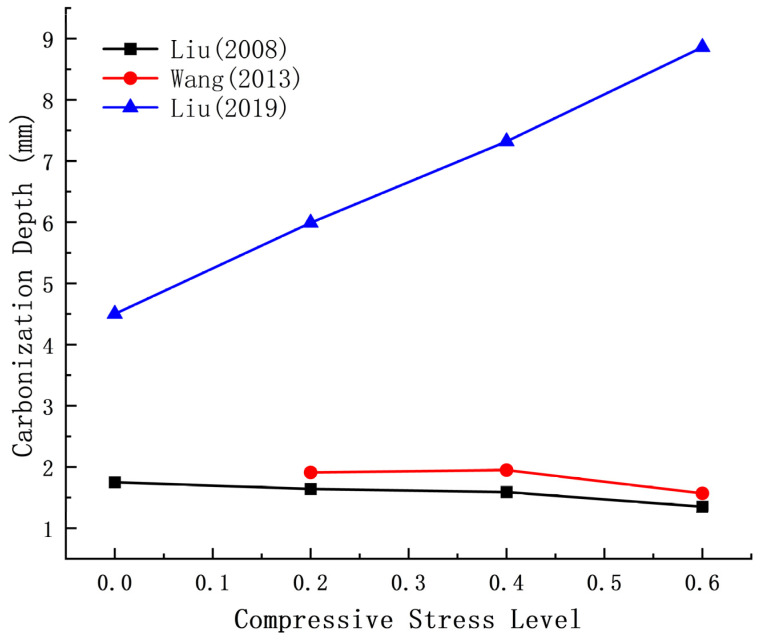
Relationship between bending stress and carbonation depth [48,87,88].

The effect of bending stress on concrete carbonation is shown in Table 4 and Figure 9.

According to Liu [87], as bending load stress increases, the carbonation depth of concrete in the bending compression zone decreases. Bending loads can inhibit CO_2_ erosion and enhance the carbonation resistance of concrete. Conversely, Wang’s experimental results [88] indicate that the carbonation depth is greatest at 0.4 fc and decreases with increasing load. However, Liu’s findings [48] reveal that the carbonation depth of concrete increases with increasing bending and compressive stress.

## 4. Effect of Coupling Effect of Stress and Carbonization on the Durability of Concrete

Carbonation and chloride ion penetration are the two primary causes of reinforcement corrosion in reinforced concrete. However, some researchers have discovered that the coupling effect of axial compression loads in coastal areas can lead to significant corrosion of steel bars, even under conditions of low chloride ion concentration and minimal carbonation depth [89].

### 4.1. Coupling of Tensile Stress and Carbonization

#### 4.1.1. Pore Structure Changes of OPC Under the Coupling Effect of Tensile Stress and Carbonization

CAI Chuanguo [90] conducted investigations and laboratory tests on concrete structures that have been in service for 80 years, analyzing the carbonation development mechanism of the surface of aged concrete. The surface carbonation layer in high tensile stress zones develops more rapidly in-depth, and its initial strength is higher than that of the underlying concrete. Over time, the oxidation of CaCO_3_ causes the carbonation layer to transition from a brittle to a plastic state, resulting in microscopic cracks that accelerate the internal progression of carbonation.

From a microscopic perspective, Wu Xianghao [91] noted that tensile stress increases the activation energy of chemical reactions, affecting the chemical bonds in concrete subjected to external stress. The molecular bonds become more susceptible to breaking, thus promoting carbonation-induced corrosion. Additionally, tensile stress in bending beams can initiate microcracks, primarily along the interface transition zone between coarse aggregates and the mortar. As a result, CO_2_ penetrates the concrete and forms solid CaCO_3_ particles that fill the pores.

**Figure 10:**
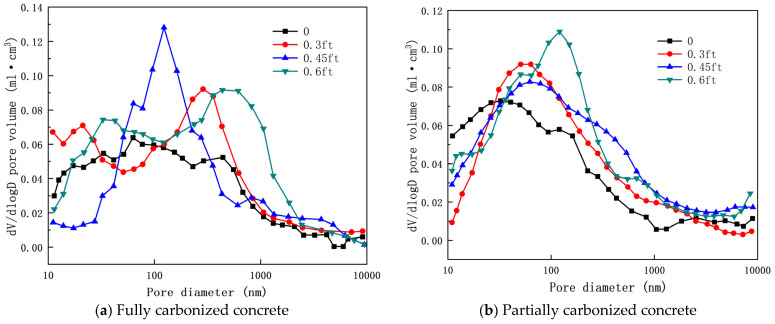
Pore size distribution curve of OPC concrete under coupling of axial tensile stress and accelerated carbonization [92].

Zhang [92] pointed out that the porosity of concrete under the combined effects of tensile stress and carbonation gradually increases with increasing tensile stress ratio. As shown in Figure 10, during the coupling process between tension and carbonation, concrete becomes increasingly loose as the tensile stress ratio rises, and the interconnection of a few cracks may lead to tensile failure of the concrete. Furthermore, microcracks in the concrete develop in the pore size range of 10,000 nm and are more prominent, with their quantity increasing as the stress ratio rises. This further accelerates the diffusion rate of CO_2_ within the concrete, decreasing its impermeability and deepening the carbonation depth.

Under the same stress ratio, the permeability resistance and carbonation depth of concrete subjected to axial tension are higher than those under axial compression and carbonation. Since the pore structure of concrete is sensitive to tensile loads, even a small number of cracks caused by tensile stress can lead to tensile failure. Consequently, new cracks begin to form within the concrete while existing cracks gradually expand, increasing pore volume.

#### 4.1.2. Pore Structure Changes of PSC Under the Coupling Effect of Tensile Stress and Carbonization

**Figure 11:**
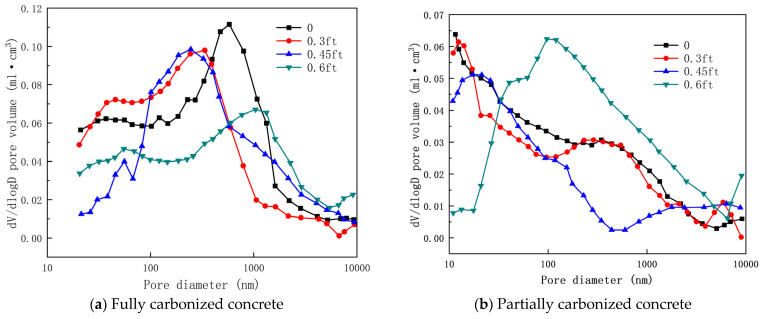
Pore size distribution curve of fly ash concrete under coupling of axial tensile stress and accelerated carbonization [92].

**Figure 12:**
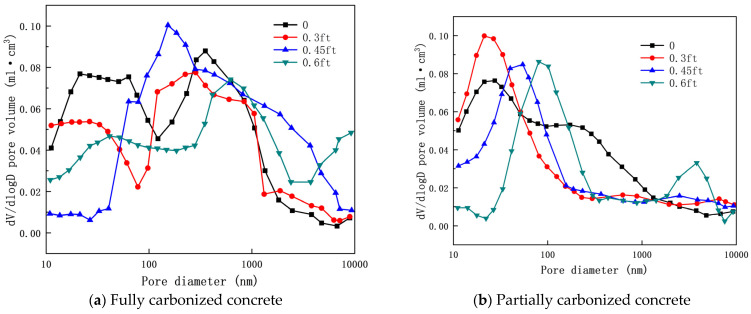
Pore size distribution curve of slag powder concrete under coupling effect of axial tensile stress and accelerated carbonization [92].

Figure 11 and Figure 12 show the carbonation pore sizes of concrete with added fly ash and slag, it can be seen that after the coupling effects of tensile stress and carbonation, the porosity of concrete containing mineral admixtures gradually increases with the tensile stress ratio. Notably, the porosity of concrete containing mineral admixtures is greater than that of OPC concrete. The damage caused by tensile stress to the pore structure of concrete containing mineral admixtures is more significant than that to OPC concrete. Under the combined effects of axial tensile stress and carbonation, changes in the pore structure of concrete are primarily influenced by pore coarsening and the rupture of pore walls.

Incorporating mineral admixtures reduces defects in the transition zone of the concrete interface, resulting in less damage from tensile stress to PSC compared to OPC concrete. Therefore, the addition of mineral admixtures enhances the pore structure of concrete, improves its gas permeability resistance, and ultimately increases the durability of concrete under the combined effects of axial tension and carbonation while reducing the amount of cement used.

### 4.2. Coupling of Axial Compression and Carbonization

#### 4.2.1. Steel Bar Corrosion and Galvanic Cell Corrosion

Compared to concrete in the tensile stress zone, concrete in the compressive stress zone typically does not exhibit large cracks. This observation suggests that after becoming blunt, the steel reinforcement does not establish fixed cathodes and anodes, resulting in uniform corrosion through chemical battery processes [93]. As the iron in the steel reacts with the oxygen and water in the environment, the resulting corrosion products infiltrate the concrete’s pores, enhancing its internal compactness. Moreover, this reaction enhances the elastic modulus of the reinforced concrete composite [94].

However, the volume expansion of the corrosion products exerts tensile stress on the surrounding concrete, leading to the formation of cracks. This process may lead to macroscopic cracks on the concrete surface, thereby reducing the overall stiffness of the structural components [95]. Additionally, the electrochemical corrosion of steel can transition from micro-cell corrosion to macro-cell corrosion. Furthermore, harmful environmental agents can penetrate the reinforcement’s surface through rust and expansion cracks, rapidly accelerating the corrosion rate and promoting further crack propagation, significantly impacting the durability of the reinforced concrete structure [96].

#### 4.2.2. Influence of Coupling Effect of Axial Compression Load and Carbonization on Gas Permeability

Sun et al. [45] found that axial compression loads induce changes in the internal microstructure of concrete compared to conditions without load. Under small loads, the primary micro defects in the concrete were partially repaired, resulting in increased resistance to gas permeability. However, further load increases promoted the re-propagation of closed microcracks and the formation of new microcracks and other defects, which eroded the concrete matrix and reduced gas permeability.

**Figure 13:**
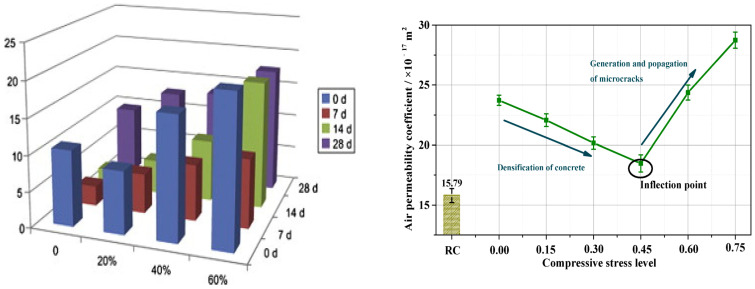
Gas permeability coefficient of concrete under coupling of axial compression load and carbonization [45,97].

Additionally, concrete carbonation, which involves the reaction of calcium hydroxide with water and carbon dioxide to produce calcium carbonate, has a beneficial physical filling effect. This process can reduce the porosity of hardened concrete and enhance its compactness and gas permeability, thereby improving the material’s porosity characteristics. As shown in the left panel of Figure 13, under constant loading conditions, the gas permeability coefficient of concrete decreases with prolonged carbonation time. When the load reaches 60% of the ultimate load, the gas permeability coefficient of the concrete is significantly higher than that of ordinary concrete.

Zhang et al. [97] employed the Autoclam method and mercury intrusion porosimetry (MIP) to assess the air permeability coefficient (kAu) and the pore structure of concrete subjected to uniaxial compression and carbonation. As shown in the right panel of Figure 13, their microscopic analysis revealed that the permeability coefficient of concrete under carbonation initially decreased with increasing stress levels but began to rise sharply once the compressive stress level exceeded 0.45.

#### 4.2.3. Influence of Axial Compression Load and Carbonization Coupling on Water Permeability

**Figure 14:**
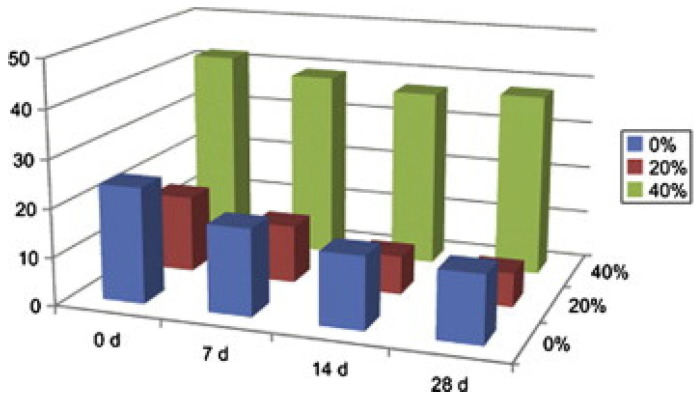
Air permeability coefficient of concrete under different stress levels [45].

Sun et al. [45] found that under constant loading conditions, the water seepage depth decreases with the carbonation time extension. Carbonation reduces the porosity of hardened concrete slurry through the physical filling and chemical compaction of carbonation products, thereby improving its compactness and reducing water permeability. As can be seen in Figure 14, when the axial load reaches 60% of the ultimate load, the water seepage depth is significantly greater than that of ordinary concrete.

**Figure 15:**
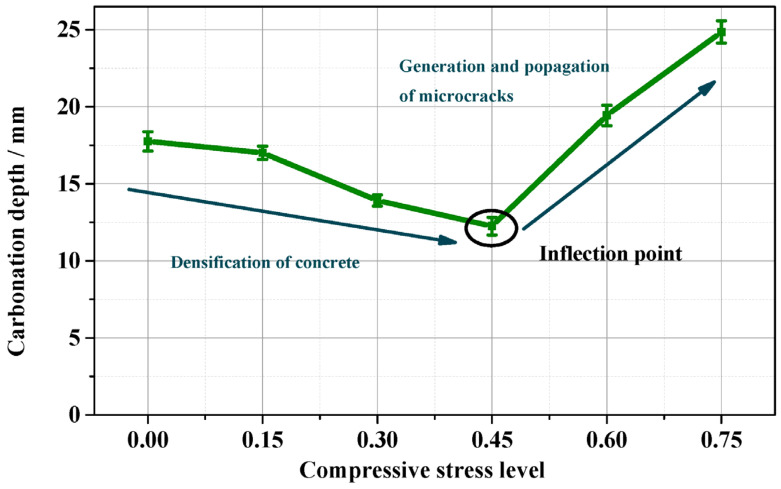
Carbonation depth of concrete under different compressive stress levels [97].

Zhang et al. [97] employed the Autoclam method and mercury intrusion porosimetry (MIP) to measure the air permeability coefficient (kAu) and pore structure of concrete subjected to uniaxial compression and carbonation, analyzing the results from a microscopic perspective. As can be seen in Figure 15, their findings indicated that the porosity of concrete under compression and carbonation initially decreases slightly but then increases rapidly as the stress level rises. This initial decrease in porosity at low stress levels reflects an increase in compactness, while the subsequent reduction in compactness at high stress levels occurs beyond a critical point of 0.45.

(11) $$\mathrm{Ca}{\left(\mathrm{OH}\right)}_{2}+{\mathrm{CO}}_{2}\to {\mathrm{CaCO}}_{3}+H_{2}O$$

(12) $${\mathrm{Ca}}_{9}H_{2}{\mathrm{Si}}_{6}O_{18}{\left(\mathrm{OH}\right)}_{8}\cdot 6H_{2}O+9{\mathrm{CO}}_{2}\to 9{\mathrm{CaCO}}_{3}+6{\mathrm{SiO}}_{2}+11H_{2}O$$

**Figure 16:**
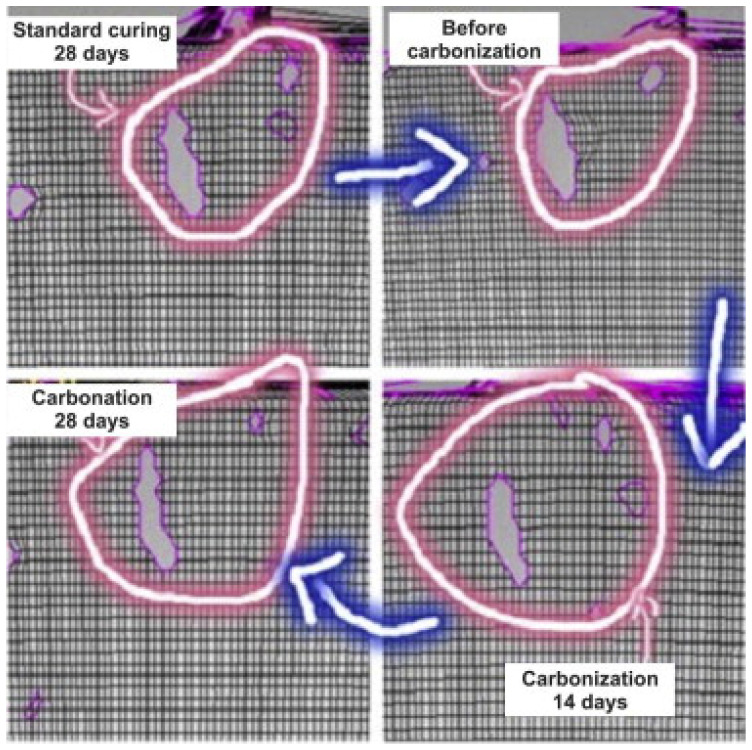
Laser scanning image of concrete after carbonization and cutting under 20% ultimate load [45].

**Figure 17:**
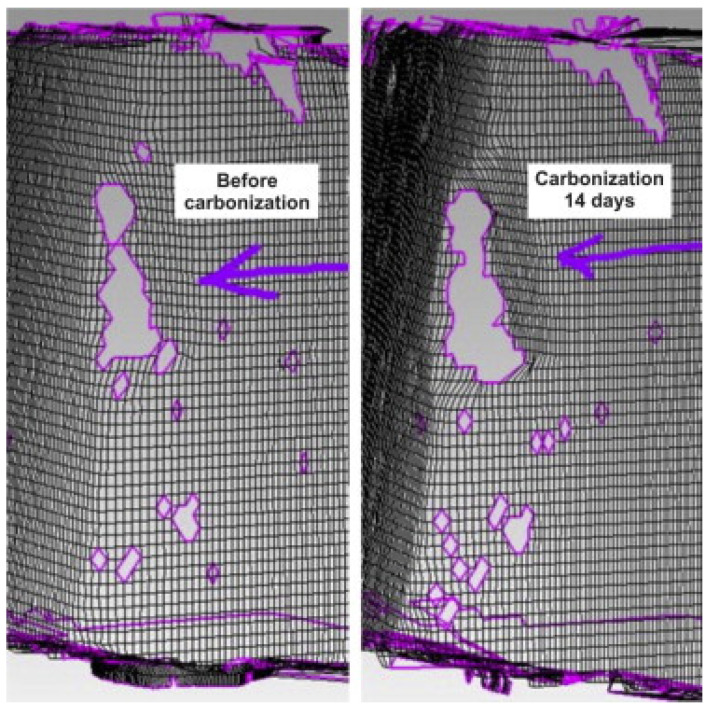
Laser scanning image of concrete cut under 60% ultimate load and carbonization [45].

As shown in Figure 16 and Figure 17, Sun et al. [45] found that when a 20% ultimate load is applied to concrete, the carbonized concrete exhibits fewer defects compared to the uncarbonized concrete. However, when subjected to a 60% ultimate load, the concrete demonstrates more defects than it does under a 20% load and than those of the benchmark untreated concrete. Specifically, at this 60% load, carbonized concrete exhibits significantly more defects than its uncarbonized counterpart. This indicates that concrete exhibits complex defects under the combined influence of varying axial compression loads and levels of carbonation; with small applied loads, microcracks in the concrete decrease as carbonation age increases, while larger loads initially reduce microcracks before causing an increase.

Zhang et al. [97] observed that the responses of kAu (permeability coefficient) and DC (carbonation depth) to stress levels followed a concave parabolic trend. At a stress level of 0.45, both kAu and DC reached their minimum values, and further increases in stress led to enhanced concrete deterioration. For stress levels below 0.45, the dual effects of low compression compaction and pore filling from carbonation products predominated, enhancing the compactness of the concrete matrix. Conversely, when the stress level exceeds 0.45, the positive influence of pore filling diminishes, while the combined effects of high compression and the disintegration of C-S-H accelerate structural deterioration. The three parameters most strongly correlated with the kAu of carbonated concrete are porosity, the ratio of transition pore volume (PROPT), and the ratio of median pore size to volume (dM-V). These parameters significantly influence permeability, and regression analysis indicates a strong linear relationship between permeability and these pore structure metrics.

### 4.3. Coupling of Bending Load and Carbonization

#### Influence of Flexural Tensile Stress and Carbonization Time Coupling Effect on Concrete Durability

**Figure 18:**
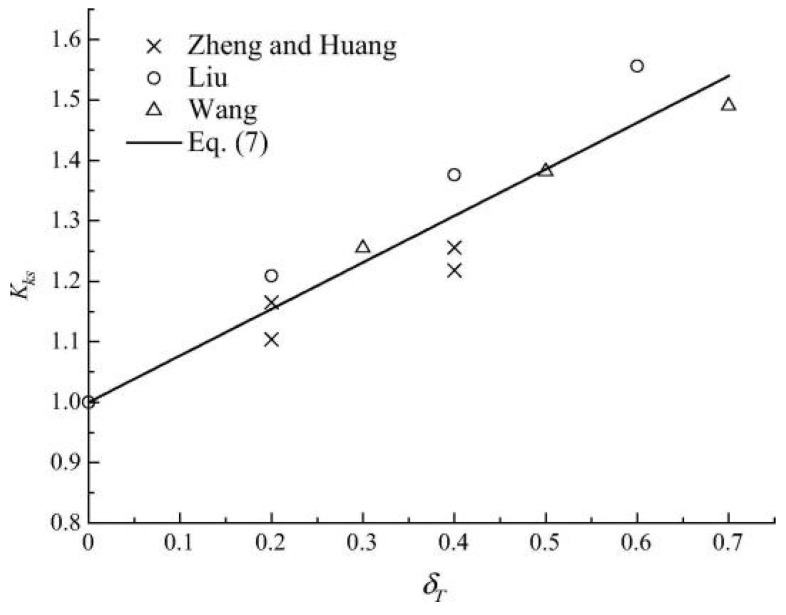
Relationship between stress influence coefficient and 7-day flexural and tensile stress level [98].

**Figure 19:**
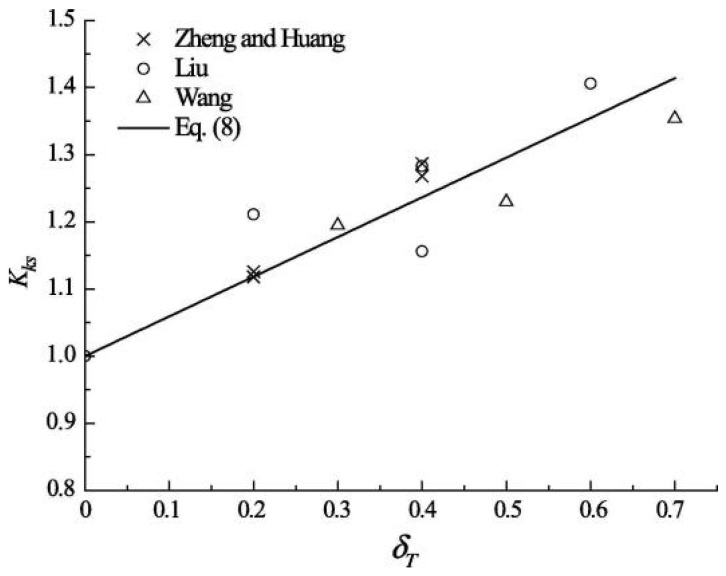
Relationship between stress influence coefficient and 14-day flexural and tensile stress level [98].

Wang et al. [98] investigated the effects of the coupling between flexural and tensile stress and carbonation time on the carbonation depth of concrete. They established a stress influence coefficient model that incorporates the effects of carbonation time, utilizing existing test data. The study found that the carbonation depth of concrete increases with higher flexural stress levels. Under the influence of flexural and tensile stress, the original micropores and microcracks in the concrete expand and widen. In contrast, new microcracks develop, leading to greater connectivity of internal micropores and an increased diffusion pathway for CO_2_. Analysis of various researchers’ results revealed that the stress influence coefficient decreases as carbonation time increases, given the same bending and tensile stress levels, Figure 18 and Figure 19 depict the relationship between the 7 d and 14 d stress influence coefficient and stress levels.

Among them: Zheng and Huang [42]; Liu [87]; Wang [84]; Equation is the data simulated by the model.

**Figure 20:**
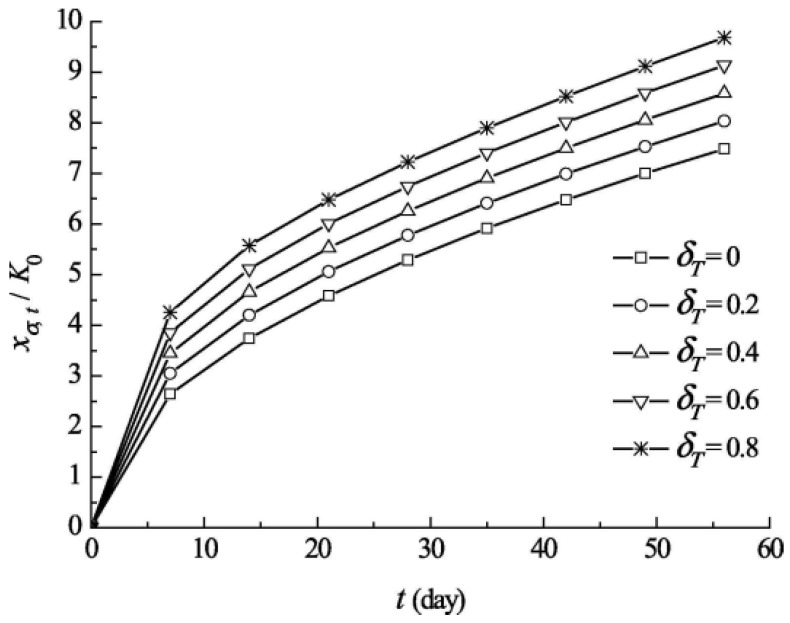
Relationship between carbonization depth, flexural and tensile stress level, and carbonization time [98].

As illustrated in Figure 20, when the carbonation duration increases from 7 to 56 days, the carbonation depth of concrete increases by 2.83, 2.64, 2.49, 2.37, and 2.28 times at flexural and tensile stress levels of 0, 0.2, 0.4, 0.6, and 0.8, respectively. Conversely, as the flexural-tensile stress level varies from 0 to 0.8, the carbonation depth of concrete also increases with carbonation times of 7, 14, 21, 28, 35, 42, 49, and 56 days, with increases of 1.61, 1.49, 1.41, 1.37, 1.34, 1.32, 1.30, and 1.29 times, respectively. These results indicate a clear coupling effect between flexural-tensile stress and carbonation time on carbonation depth.

### 4.4. Coupling of Cyclic Load and Carbonized Concrete

Saito and Imai [99] tested 80 specimens under uniaxial cyclic tensile stress specimens, with minimum stress levels set at 8% and maximum stress levels at 75.0%, 77.5%, 82.5%, 85.0%, and 87.5%. Throughout the loading cycles, the elastic modulus remained relatively stable until failure, irrespective of the stress level, while the residual strain significantly increased during loading. Lei et al. [27] found that the elastic recovery rate decreases as the stress level increases, indicating that higher compressive stress levels result in greater plastic deformation and deterioration. Additionally, the elastic recovery rate increases with higher levels of concrete strength, the relationship between compressive stress level and elastic recovery is shown in Table 5

**Figure 21:**
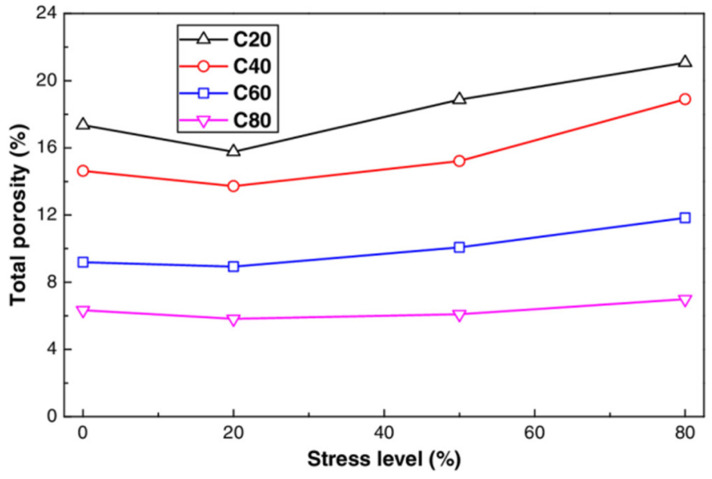
Relationship between stress level of concrete and total porosity after cyclic compression load [27].

**Figure 22:**
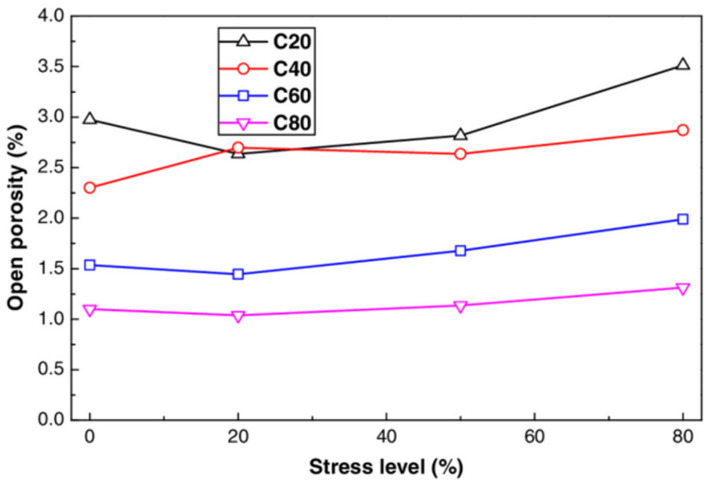
Relationship between stress level and porosity of concrete under cyclic compression load [27].

Figure 21 and Figure 22 illustrates the relationship among compressive stress level, total porosity, and open porosity in concrete following cyclic compression loading. As the strength of concrete increases, both total porosity and open porosity decrease. When the stress level is below 20%, porosity continues to decline; however, beyond this threshold, both porosity types generally increase with rising compressive stress. Notably, the open porosity of C40 concrete continues to increase throughout this range. This indicates that when the applied load is less than 20% of the peak stress, the compressive load compacts the microstructure of the concrete. Conversely, exceeding 20% of the peak stress results in the formation of microcracks within the concrete.

**Figure 23:**
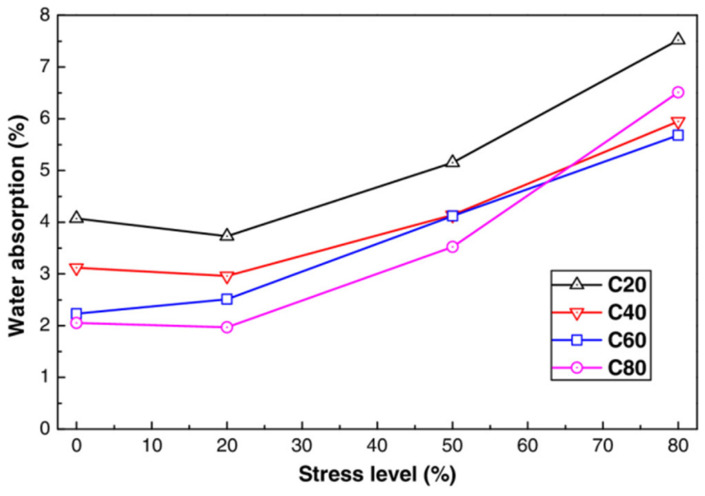
Connection between stress level and water absorption in concrete [27].

As shown in Figure 23, When the compressive stress level is below 20%, water absorption initially decreases; however, beyond this threshold, water absorption increases. This trend aligns with the changes in concrete porosity under varying compressive loads, suggesting that the applied load stress can compact the original pores in concrete. Once the threshold stress level is surpassed, microcracks form and propagate, enlarging the crack openings and increasing connectivity. The compacted microstructure of high-strength concrete results in reduced water absorption as the strength increases. For C60 concrete, the water absorption rate steadily increases with stress levels, whereas for C80 concrete, it rises sharply after reaching 50% of the stress level due to microcrack formation. These cracks can lead to substantial durability issues for high-strength concrete under high load conditions.

### 4.5. Steel Bar Corrosion Under the Coupling Effect of Static Load and Carbonization

**Figure 24:**
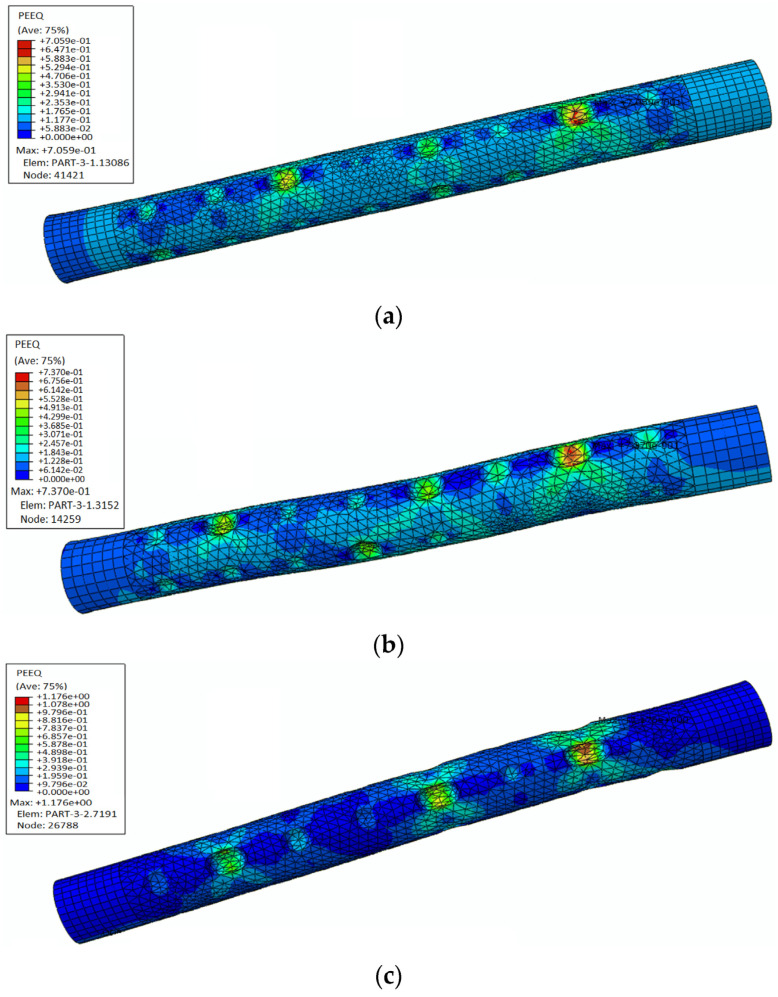
Finite element simulation results: (**a**–**c**) are three steel bar models respectively [100].

Xu [100] employed two parameters—the area box value and the arithmetic mean deviation—to characterize the corrosion morphology and pitting distribution observed in experiments. The employed indicates that static loading significantly influences the corrosion characteristics of steel bars. Local stress concentrations induced by tensile stress lead to the formation of more irregularly shaped rust pits. The stress-strain curves of steel bars subjected to the combined effects of carbonation and static loading reveal that the latter notably influence corrosion behavior. Specifically, this coupling facilitates the growth of corrosion pits along preferred orientations, resulting in more angular pit shapes and greater non-uniformity in their depth distribution. Finite element analysis confirmed that the presence of corrosion pits induces stress concentrations in the corroded reinforcement bars. The influence of various factors can be ranked as follows: pit depth > pit shape > corrosion mass loss ratio > error. Notably, both pit depth and shape emerged as significant factors influencing the maximum equivalent plastic strain, Figure 24 shows the finite element simulation of rebar corrosion.

## 5. Impact of Various Additives on the Carbonation Depth of Concrete

Song et al. [101] reported a significant reduction in the capillary water absorption capacity of carbonized concrete, which decreased by 20.0–26.5%, while the capillary water absorption coefficient declined by 30.8–37.8%. Compared to uncarbonated concrete, the maximum pore size in carbonized concrete decreased by 13.9–15.1 nm, reflecting a maximum reduction of 41.8%. This trend suggests that a higher water-cement ratio exacerbates the deterioration of the pore structure, potentially affecting the overall durability of the concrete. Zhang [39] found that, for the same carbonation duration, the average promotion rates of carbonation depth for concrete incorporating fly ash at contents of 5%, 10%, 20%, and 30% were 6.4%, 14.9%, 59.8%, and 73.5%, respectively. This highlights the critical role of fly ash in modifying carbonation behavior; however, its interaction with the carbonation process raises concerns regarding durability. The carbonation of fly ash and Portland cement concrete severely impacts durability, prompting various researchers to explore mitigation strategies [102,103]. Liu [104] examined concrete mixtures containing single fly ash, single desert sand, and combinations of both, finding that the carbonation depth initially decreased and then increased with the desert sand substitution rate, achieving a minimum of 20% substitution. The minimum carbonation depth occurred with 10% fly ash and 20% desert sand in the combined mixture, suggesting that careful selection of materials is essential for optimizing carbonation resistance. Chen [105] established a carbonation model applicable to ordinary concrete mixed with fly ash, slag, or both, emphasizing the necessity of careful selection for optimizing carbonation resistance. Bai [106] investigated the effects of various mixing methods on the carbonation resistance of concrete using rapid carbonation tests. The findings indicated that a single mixing of regenerated micro powder outperformed a single mixing of fly ash at the same substitution rate. This suggests that innovative mixing techniques can significantly enhance the durability of concrete.

Furthermore, combining recycled micro powder, fly ash, and silica fume significantly improved carbonation resistance, achieving a maximum carbonation depth of 14.6 mm at a 40% replacement rate. Huang et al. [107] modified the water absorption and pore structure of bulk fly ash cement paste by incorporating nano-silica. Win et al. [108] investigated the synergistic effects of graphene nanosheets and fly ash on the mechanical properties and microstructure of calcium aluminate cement composites, revealing the potential of advanced materials to revolutionize concrete performance. Zheng [51] enhanced concrete by adding fibers mixed with iron tailings, discovering that performance improved with increasing tailings content up to a threshold, after which it declined; the addition of fibers effectively enhanced strength, elastic modulus, peak strain, and carbonation depth.

Collectively, these studies underscore the substantial impact of various materials on concrete performance and durability. However, the pursuit of more effective and environmentally friendly alternatives remains an ongoing challenge, underscoring the need for continued innovation in material science to enhance the sustainability of concrete structures.

## 6. Discussions

This paper aims to provide insights and references for researchers in concrete durability and materials science by reviewing a comprehensive body of research on stress and concrete carbonation while also offering theoretical guidance for engineering practice.

1. Literature Screening: We prioritized the screening process based on relevance. Initially, we selected highly relevant studies and organized them according to their impact factor, journal ranking, and publication date, ensuring that the cited works were high-quality and closely related to our topic. Additionally, we conducted supplementary searches using targeted keywords for specific research areas that require further exploration.
2. Content Summary: As a review paper, we focused on synthesizing and distilling different studies’ experimental processes and findings, including model proposals and validations. We also examined modifications to these models and presented contrasting perspectives on similar issues, thereby allowing readers to gain a more comprehensive understanding.
3. Data Visualization: For figures, we recreated graphs using data extracted from various studies to enhance clarity. We made every effort to maintain visual clarity and data accuracy, particularly for 3D graphs where data extraction presented challenges.
4. Selection of Representative Studies: For findings reported across multiple studies, we selected representative papers for detailed discussion, while other supporting studies were referenced but not elaborated upon.

Through our analysis of the existing literature, we found that while some progress has been made in understanding the effects of carbonation on reinforced concrete, research on durability and lifespan prediction under the combined effects of stress and carbonation remains in its early stages. Current challenges include:

1. The increasing trend of utilizing mineral admixtures to partially replace Portland cement for CO_2_ emission reduction, yet the carbonation behavior of such concrete under stress conditions is not fully understood;
2. The dynamics of CO_2_ transport vary significantly under stress, necessitating investigation into the coupling effects between load and carbonation;
3. Existing prediction models for carbonation depth often fail to integrate the chemical factors from the carbonation reaction with physical stress factors; a comprehensive approach should include CO_2_ transport rates and carbonate content to improve predictive accuracy under load;
4. Accelerated carbonation tests frequently employ CO_2_ concentrations significantly higher than those found in natural environments, raising questions about the applicability of these models to real-world scenarios and highlighting the need for more effective testing devices for rapid carbonation assessments.

For future research, we recommend the following specific methodologies:

1. Development of standardized testing protocols for carbonation assessment that can be universally applied across studies, ensuring consistency and comparability of results;
2. Implementation of innovative modeling techniques incorporating multi-physics simulations to better understand the coupled effects of environmental factors and mechanical stresses on carbonation processes;
3. Exploration of real-time monitoring technologies to track carbonation progression in situ, providing valuable data for predicting service life and durability;
4. Investigation into the long-term effects of varying loading conditions on the carbonation behavior of concrete containing different mineral admixtures to establish comprehensive guidelines for sustainable concrete design.

## 7. Conclusions

Concrete carbonation significantly influences durability, with extensive research identifying key factors such as the water-cement ratio, relative humidity, and temperature. Generally, within specific humidity and temperature ranges, an increase in relative humidity correlates with a reduction in carbonation depth, while elevated temperatures tend to enhance carbonation values. Macroscopically, higher tensile stress accelerates the carbonation rate. Regarding compressive stress, some scholars propose a critical threshold at 0.2 fc, below which carbonation depth diminishes and beyond which it increases; others suggest a threshold at 0.3 fc. Enhanced flexural compression reduces carbonation depth, while increased flexural tension has the opposite effect.

Microscopically, the coupling of axial tension and carbonation compromises permeability and enlarges pore volume, adversely affecting durability. Conversely, axial compression may lead to steel corrosion, which may enhance gas and water permeability. Bending loads refine the pore structure, while cyclic loads induce significant plastic deformation, resulting in increased total and open porosity, ultimately leading to steel corrosion. Although substantial progress has been made in understanding the impact of carbonation on reinforced concrete, there remains a need for further research into durability and life prediction models that take into account the combined effects of stress and carbonation. Developing standardized testing methods and advanced modeling approaches to address these gaps is expected to provide valuable insights for enhancing the longevity of concrete structures under complex loading and environmental conditions.

## Figures and Tables

**Table 1:** Correlation between water-cement ratio and concrete carbonation.

Concrete	FA (%)	Water Ash Ratio	Carbonization Depth (mm)					References
			3 d	7 d	14 d	28 d	60 d	
OPC	0	0.45		0.12	0.41	0.48	0.68	[58]
	15	0.45		0.92	1.45	1.78	2.35	
	30	0.35		0.74	1.13	1.56	1.85	
		0.45		1.67	2.78	3.55	4.38	
		0.55		2.89	4.02	5.89	7.75	
OPC	20	0.4	0.1	0.2	0.3	0.4		[57]
		0.45	3	3.4	4.3	4.8		
		0.5	12.3	17.1	25.3	30		
		0.55	8.8	10.8	15.3	17.2		
	30	0.4	0.6	0.9	2.2	4.3		
		0.45	4.6	5.3	5.9	6.4		
		0.5	8.3	8.7	9.5	11.6		
		0.55	9.6	12.9	14.2	14.5		
	40	0.45	8.6	12.1	13.3	17.8		
		0.5	9.9	14.8	15.3	17.3		
		0.55	12.2	15.8	18.5	27.3		
	50	0.5	11.8	15	18.9	27.6		
		0.55	14.8	18.8	21.1	25.6		
	60	0.5	30	33.2	50	50		
		0.55	30	50	50	50		
PSC	0	0.35	0	0	0	0		[57]
		0.4	0	0	0	0		
		0.45	0.5	1.9	2.9	3.8		
		0.5	3.1	4.5	4.6	4.8		
	20	0.4	4.1	4.5	5.1	5.7		
		0.45	7.3	8.7	9.5	10.1		
		0.5	9.7	11.1	11.7	12.9		
	40	0.4	6.8	7.7	8.3	8.8		
		0.45	7.6	9.7	10.6	12.1		
		0.5	10.3	11.6	13.3	15.2		
PSC	7	0.33	4.7	5.3	5.8	6.4		[59]
		0.35	5.9	6.5	7.1	7.6		
		0.38	7.6	8.8	10.3	11.4		
		0.49	8.8	11.6	14.1	18		
OPC	0	0.4	0	2.4	2.9	3.3		[60]
		0.5	3.1	3.9	3.9	8		
		0.6	3.2	9.1	10.9	16		

**Table 2:** Effect of compressive stress level on concrete carbonization depth.

Researcher	Compressive Stress	Carbonation Depth (mm)	References
Zhang	0	25.45	[81]
	0.3	23.2	
	0.5	22.5	
	0.7	19.95	
Shi	0	14.72	[82]
	0.15	13.56	
	0.3	12.7	
	0.45	11.6	
	0.6	10.4	
	0.75	7.9	
Wu	0	6.57	[83]
	0.05	5.71	
	0.3	2.75	
	0.5	1.35	
Wang	0	2.71	[84]
	0.2	2.35	
	0.35	2.21	
	0.5	1.92	
Luo	0	11.85	[85]
	0.15	10.1	
	0.3	8.75	
	0.45	7.06	
	0.6	5.21	
	0.75	1.36	
Sun	0	4.16	[78]
	0.3	3.91	
	0.5	3.7	
Zhang	0	17	[72]
	0.25	16	
	0.5	15.01	
	0.75	15.61	
Xu	0	1	[77]
	0.1	0.87	
	0.3	0.78	

**Table 3:** Effect of flexural tensile stress on concrete carbonization depth.

Researcher	Tensile Stress	Carbonation Depth (mm)	References
Zhang	0	29	[81]
	0.3	29.6	
	0.5	30.2	
	0.7	31	
Shi	0	14.72	[82]
	0.15	15.1	
	0.3	15.82	
	0.45	16.76	
	0.6	17.9	
	0.75	19.1	
Wu	0	6.22	[83]
	0.05	6.89	
	0.3	7.28	
	0.5	7.93	
Zhang	0	8.5	[86]
	0.2	9.1	
	0.35	10	
	0.5	11.4	
	0.65	12.8	
	0.8	14	
Liu	0	1.12	[87]
	0.2	1.59	
	0.4	2.16	
	0.6	2.56	
Wang	0	2.71	[84]
	0.3	2.87	
	0.5	3.12	
	0.7	3.42	
Luo	0	11.86	[85]
	0.15	12.41	
	0.3	13.54	
	0.45	15	
	0.6	16.8	
	0.75	18.57	
Sun	0	4.20	[78]
	0.15	4.59	
	0.3	4.89	
Zhang	0	16.96	[72]
	0.25	19.08	
	0.5	20.17	
	0.75	24	
Wang	0.2	3.73	[88]
	0.4	4.04	
	0.6	4.38	
Liu	0	4.74	[48]
	0.2	4.56	
	0.4	4.01	
	0.6	3.55	
Xu	0	1	[77]
	0.2	1.23	
	0.4	1.62	
	0.6	1.65	
	0.8	1.69	

**Table 4:** Effect of bending compressive stress on concrete carbonization depth.

Researcher	Compressive Stress	Carbonation Depth (mm)	References
Liu	0	1.75	[87]
	0.2	1.64	
	0.4	1.59	
	0.6	1.35	
Wang	0.2	1.91	[88]
	0.4	1.95	
	0.6	1.57	
Liu	0	4.5	[48]
	0.2	5.99	
	0.4	7.32	
	0.6	8.86	

**Table 5:** Elastic recovery rate of coagulation ten at 120 s duration under different compressive stress levels [27].

Concrete	Compressive Stress Level				
	20%	40%	50%	60%	80%
C20	23.26	17.58	15.32	12.99	10.82
C40	31.38	24.26	17.15	15.33	12.32
C60	40.43	32.49	24.87	21.01	19.32
C80	65.34	50.65	46.57	43.18	40.26
